# Multifunctional MXene/CNTs Composite Fibers with High Strength, Conductivity, and Bending Sensitivity Fabricated via Sodium Alginate-Assisted Wet Spinning

**DOI:** 10.3390/bios16100532

**Published:** 2026-09-24

**Authors:** Ting Zheng, Shuting Xu, Zherui Zhang, Kaixuan Wang, Xiaodong Wang, Guoxing Sun

**Affiliations:** 1 Joint Key Laboratory of the Ministry of Education, Institute of Applied Physics and Materials Engineering, University of Macau, Macau SAR 999078, China; tingzheng@um.edu.mo; 2 Key Laboratory of Superlight Materials and Surface Technology, Ministry of Education, College of Materials Science and Chemical Engineering, Harbin Engineering University, Harbin 150001, China

**Keywords:** MXene, carbon nanotubes, sodium alginate, wet-spinning method, multicomponent composite fibers

## Abstract

Multicomponent nanocomposite fibers with synergistic properties are highly desirable for next-generation flexible electronics and sensors. In this study, MXene, carbon nanotubes (CNTs), and their composite fibers were successfully fabricated via a wet-spinning method using sodium alginate (SA) as a binder and fiber-forming agent. The morphology, mechanical properties, electrical conductivity, electrothermal behavior, and bending sensing performance of the resulting fibers were systematically investigated. All fibers exhibited longitudinal surface wrinkles and a nearly circular cross-section, indicating good process controllability. The MXene/CNTs@SA fibers, featuring a hierarchical synergistic network constructed from one-dimensional CNTs and two-dimensional MXene nanosheets, demonstrated significantly enhanced mechanical performance, with a tensile strength of 76.38 ± 7.09 MPa and conductivity of 16.14 ± 0.36 S·cm^−1^. Under a 12 V applied voltage, the MXene/CNTs@SA fibers achieved a saturation temperature of 52.77 °C and a power density of 2.95 kW·cm^−3^, demonstrating balanced electrothermal performance. Furthermore, the MXene/CNTs@SA fibers exhibited excellent flexibility and sensing stability, with resistance variations within ± 1.6% over repeated 100 bending–release cycles. The bending gauge factor was determined to be 7.36, confirming high sensitivity and cyclic durability. These results demonstrate that the wet-spun MXene/CNTs@SA composite fibers, with their robust mechanical strength, enhanced electrical conductivity, reliable electrothermal response, and superior bending sensitivity, hold great promise for applications in flexible electronic devices and wearable sensors.

## 1. Introduction

With the rapid development of flexible wearable electronics and human–machine interaction technologies, next-generation conductive materials are required not only to possess high electrical conductivity and mechanical strength but also to urgently integrate multiple functionalities such as electrothermal conversion and deformation sensing [1,2,3].

MXene, a novel two-dimensional (2D) material, has attracted considerable attention since its discovery by Yuri Gogotsi’s group in 2011 [4]. Owing to its high metallic conductivity, excellent mechanical properties, abundant surface functional groups, and large interlayer spacing [5,6], MXene has found widespread applications in energy storage, electronics, electromagnetic interference shielding, sensors, actuators, environmental remediation, and biomedicine [7,8,9]. However, most MXene-based applications have been realized primarily in the form of powders or thin films [10,11]. To fully exploit the intrinsic properties of MXene at the macroscopic scale, assembling MXene nanosheets into macroscopic fibers offers an effective strategy—not only mitigating the severe agglomeration issues commonly encountered in powder form but also significantly expanding its application horizon [12,13]. Benefiting from the abundant hydrophilic groups (–OH, –O, –F) on MXene surfaces, aqueous MXene dispersions are readily processable into fibrous architectures. Consequently, MXene fibers inherently exhibit high specific modulus, high specific strength, good flexibility, excellent electrical conductivity, and outstanding electrochemical performance, rendering them highly promising for smart electronics, wearable devices, flexible sensors, and energy storage systems [14,15]. Since 2017, various methods including wet-spinning, coating, and electrospinning have been developed to fabricate MXene fibers [16,17,18]. Nevertheless, the preparation—and particularly the scalable industrial production—of MXene fibers still faces significant technical bottlenecks and challenges. Key issues such as non-uniform lateral size distribution of MXene nanosheets, weak interlayer interactions, and insufficient self-supporting structural stability directly limit the continuous spinnability and overall performance of pristine MXene fibers. Specifically, pure MXene fibers often suffer from unsatisfactory mechanical strength, limited electrical conductivity, and poor sensing performance, which severely restrict their practical applications.

To address these challenges and improve the fiber formability and overall properties, incorporating auxiliary components into MXene-based systems has emerged as a promising strategy. Carbon nanotubes (CNTs), as quintessential one-dimensional (1D) nanomaterials, possess extraordinary mechanical properties, excellent electrical conductivity, and remarkable flexibility, making them ideal reinforcing materials for high-performance fibrous materials [19,20]. Notably, the assembly of CNTs into macroscopic fibers has been extensively investigated since their discovery, with a variety of fabrication techniques—including wet-spinning, dry-spinning, and array-spinning—having been well established [21,22]. Compared to MXene, CNTs are intrinsically more amenable to fiber formation due to their high aspect ratio, flexible tubular structure, and strong inter-tube van der Waals interactions, which collectively facilitate continuous spinnability and structural integrity [23,24]. In the context of MXene-based fiber fabrication, the introduction of CNTs can act as conductive bridges interconnecting adjacent MXene nanosheets, effectively inhibiting the restacking of 2D MXene layers and constructing a highly robust hierarchical conductive network that facilitates efficient charge transport. Second, CNTs function as mechanical skeletons within the fiber matrix, efficiently transferring and distributing loads during deformation, alleviating stress concentration, and thereby significantly enhancing the tensile strength and structural stability of the composite fibers. Furthermore, the synergistic interplay between CNTs and MXene—arising from hydrogen bonding and π–π interactions between the abundant functional groups on MXene surfaces and the CNT frameworks—promotes strong interfacial adhesion, which is essential for maintaining structural integrity under repeated mechanical stress [25,26]. 2D MXene nanosheets, with their hydrophilic surfaces, high metallic conductivity, and abundant terminal functional groups, can provide excellent conductive pathways and interfacial interaction sites. Collectively, the integration of CNTs into MXene fiber systems simultaneously addresses poor spinnability, insufficient electrical conductivity, and inadequate mechanical properties of MXene fibers using a single nanomaterial additive, offering a promising pathway toward the development of high-performance, multifunctional fibrous materials for flexible electronics and wearable applications. Last but not least, developing a facile and scalable method to fabricate MXene/CNT composite fibers that preserve the intrinsic advantages of each component while exhibiting a well-defined synergistic structure remains an unmet challenge. Existing fabrication routes, such as dry-spinning or chemical vapor deposition (CVD)-based methods, often involve complex equipment, harsh conditions, or toxic solvents, severely restricting their practical application.

In this work, a green wet-spinning strategy uses biocompatible sodium alginate (SA) as both a binder and a fiber-forming matrix to successfully fabricate MXene/CNTs (MXene/CNTs@SA) composite fibers. This design fully capitalizes on the synergistic interplay between CNT and MXene, collectively constructing a highly interconnected hierarchical electron transport network. A comparative investigation was conducted on the morphology, mechanical properties, electrical conductivity, and electrothermal characteristics of pristine MXene fibers, CNTs fibers, and MXene/CNTs@SA composite fibers. Furthermore, the sensing performance of the MXene/CNTs@SA fibers was systematically evaluated through repeated bending–release cycle tests and bending sensitivity measurements. The resulting fibers exhibit robust mechanical properties, stable electrothermal response, and sensitive bending perception capabilities, endowing the MXene/CNTs@SA composite fibers with great potential for diverse applications, including flexible electronic devices, wearable thermal management, and motion detection.

## 2. Materials and Methods

### 2.1. Materials

The Ti_3_AlC_2_ MAX phase powder (99% purity), multi-walled carbon nanotubes (MWCNTs, ≥98% purity, 10–20 nm diameter, 10–30 μm length), and sodium alginate (SA, food grade) were commercially obtained from Jinlei Nanotechnology (Ningbo, China), Chengdu Zhongke Times Nano (Chengdu, China), and Qingdao Mingyue Seaweed Group (Qingdao, China), respectively. Anhydrous CaCl_2_ and HCl were purchased from Hengxing Reagent (Tianjin, China). All reagents were used as received without additional treatment.

### 2.2. Preparation of MXene

MXene was fabricated via LiF–HCl etching of MAX. Briefly, LiF (1 g) dissolved in 9 M HCl (25 °C, 500 rpm, 15 min) was mixed with MAX (1 g) and reacted at 35 °C for 24 h. The product was rinsed with 1 M HCl, centrifuged with H_2_O at 3500 rpm to pH ≥ 6, re-dispersed, sonicated (ice bath, 1 h), and centrifuged at 5000 rpm for 1 h. The delaminated supernatant was dried at 50 °C for 12 h to obtain MXene powder.

### 2.3. Preparation of Spinning Solution

To ensure consistent concentration, 60 mg of nanomaterials (MXene and CNTs) were dispersed in 5 mL of DI water using an ultrasonic dispersion instrument for 30 min in pulse mode (2 s on, 4 s off) to obtain a uniform nanomaterial suspension. The resulting MXene and CNTs dispersions were mixed in various weight ratios: 1:0, 0:1, and 1:1. Subsequently, sodium alginate (SA) powder was added to each mixed suspension at a SA:total solids mass ratio of 1:4 (corresponding to 20 wt% SA relative to total solid content), and the mixture underwent sequential stirring: first at 50 °C for 30 min, then at room temperature for 4 h. The resulting spinning solutions were designated as MXene@SA, CNTs@SA, and MXene/CNTs@SA, respectively. The 20 wt% SA content was selected based on preliminary optimization experiments balancing spinnability and mechanical integrity (Figure S1).

### 2.4. Wet-Spinning of Hybrid Fibers

The wet-spinning method was used to fabricate the fibers using a 10 wt% CaCl_2_ aqueous solution as the coagulation bath. The spinning dope was extruded through a syringe needle (inner diameter: 0.5 mm) at a flow rate of 1.2 mL min^−1^ into a coagulation bath that was continuously rotated at 35 rpm. After 2 h of coagulation, the fibers were immersed in ethanol for 2 h to remove surface calcium ions. The fibers were then air-dried. The final fibers were denoted as MXene@SA fibers, CNTs@SA fibers, and MXene/CNTs@SA, respectively.

### 2.5. Characterization

#### 2.5.1. Morphologies and Structure

SEM (Apreo S Lovac, Thermo Fisher Scientific, Waltham, MA, USA) was used to characterize fiber surface morphologies, with EDS for elemental mapping. Cross-sectional areas were determined via ImageJ 1.53t (National Institutes of Health, Bethesda, MD, USA) for normalization of conductivity and mechanical properties. FTIR spectra were collected on a Spectrum 3 spectrometer (PerkinElmer, Shelton, CT, USA) in the range of 4000–500 cm^−1^ at room temperature using KBr pellets as a reference.

#### 2.5.2. The Mechanial and Electrical Properties

Mechanical properties were measured using a ZQ-990L (ZHIQU Precision Instruments Co., Ltd., Dongguan, China) tester, and tensile strength (σ) was calculated as:

(1) $$\sigma =\frac{F}{A}$$

where σ is the tensile strength (MPa), F is the maximum force (N) and A is the cross-sectional area of the fibers (mm^2^).

Electrical resistance was measured by a Keysight 34465A digital multi-meter (Keysight Technologies, Santa Rosa, CA, USA). The conductivity (σ) was calculated as:

(2) $$\sigma =\frac{L}{RS}$$

where σ is the conductivity (S cm^−1^), R is the resistance (Ω), S is the cross-sectional area (cm^2^), and L is the length of the fiber (cm).

Quantitative data are presented as mean ± standard deviation (n = 3 or more). Statistical significance between two groups was determined using an unpaired two-tailed Student’s *t*-test. Significance levels are denoted as * *p* < 0.05, ** *p* < 0.01, and *** *p* < 0.001.

#### 2.5.3. Electrothermal Performance

The electrothermal performance of the fibers (length: 2 cm) was evaluated using an infrared thermographic camera (FLIR), and the surface temperature distribution was continuously recorded. Additionally, the fibers were connected to the copper wires using conductive silver adhesive to minimize the contact resistance. A DC power (12 V) was used to apply voltage across both ends of the fiber. The power density (P_S_) was calculated using Joule’s law, assuming complete conversion of electrical energy into heat:

(3) $$Ps=\frac{U^{2}}{VR}\times 10^{-3}$$

where P_S_ is the volumetric power density (kW cm^−3^), U is the applied voltage (V), R is the resistance (Ω), and V is the geometric volume of the entire fiber (cm^3^), calculated from the fiber diameter (measured by optical microscopy) and length.

#### 2.5.4. Bending Sensing Performance

The gauge factor (GF) of the composite fibers was calculated based on the relative resistance change (ΔR/R_0_) and the applied bending strain (ε),

(4) $$GF=(\Delta R/R_{0})/ℇ$$

The bending strain was derived from the curvature radius (r) and the specimen diameter (h) as below:

(5) ε=±h/2r

To obtain the curvature radius r, we used the geometric relationship between the chord length and the arc length of the bent fiber:

(6) c=2r·sin(l/2r)

where l = 55.5 mm is the fiber length (arc length), which is also the initial distance between the two fixed endpoints before bending, and c is the straight-line distance (chord length) between the two fixed endpoints after bending. The fiber diameter was measured to be h = 66.05 μm using an optical microscope (Olympus BX53M, Olympus Corporation, Tokyo, Japan) and ImageJ 1.53t software.

## 3. Results and Discussion

### 3.1. Morphology and Structural Characterization of MXene

In this work, MXene nanosheets were successfully synthesized via a LiF–HCl etching method under optimized conditions (9 M HCl, 35 °C, and 1 h sonication). The surface morphology and microstructure of the as-prepared MXene were characterized by SEM and TEM, as shown in Figure 1a,b. The SEM images reveal that the obtained MXene exhibits a typical thin-layered structure with smooth and clean surfaces, free from noticeable defects, and no granular residues are observed on the surfaces or edges, indicating that no significant oxidation occurred during etching. The high-resolution TEM image in Figure 1c further demonstrates that the MXene sheets have an average thickness of approximately 1.6 nm, confirming the successful preparation of monolayer MXene. XRD analysis (Figure 1d) was also performed to verify the phase transformation from the MAX precursor to MXene. The characteristic peaks of the MAX phase at 2θ = 9.52° (102), 39.02° (104), and 44.9° (106) disappeared after etching, while a new diffraction peak corresponding to the (002) plane of MXene appeared at 2θ = 6.38°, confirming the complete removal of the Al layer and the successful formation of MXene.

### 3.2. Morphology and Structural Characterization of MXene/CNTs@SA Fibers

To determine the appropriate SA content for MXene@SA fiber fabrication, preliminary spinning trials were conducted with SA contents ranging from 10 to 20 wt% (Figure S1). When the SA content decreased below 20 wt%, the fibers became increasingly discontinuous and difficult to collect in practical lengths, with tensile strength dropping from 67.89 ± 1.27 MPa (20 wt%) to 37.38 ± 1.37 MPa (15 wt%) and 20.60 ± 2.59 MPa (10 wt%). Although lower SA content would theoretically reduce the insulating polymer fraction, the severe loss of mechanical integrity at <20 wt% SA precluded reliable handling. Therefore, 20 wt% SA was selected as the optimal composition balancing spinnability, mechanical strength, and filler loading.

Subsequently, three different multicomponent nanocomposite fibers were successfully prepared through a stable wet-spinning method (Figure 2). The surface and cross-sectional morphologies of the three types of fibers are presented in Figure 3. Some fibers exhibit relatively flat surfaces covered with numerous particulate structures, which are presumed to be calcium ion precipitates formed in the coagulation bath [20]. Longitudinal ripples observed on the surfaces of all fiber types are likely due to the shrinkage of the gel fibers during the solidification process. The cross-sectional SEM images reveal that all fibers possess an approximately circular geometry.

As shown in the high-magnification SEM images (Figure 3a), the MXene@SA fibers exhibit a distinctive microstructure characterized by serrated, bended, and densely packed lamellar architectures, indicative of the successful assembly of MXene nanosheets within the SA matrix. In contrast, the CNTs@SA fibers (Figure 3b) display a disordered arrangement of tubular CNTs, which tend to aggregate into larger bundle-like structures along the fiber axis, consistent with previous reports [27]. For the hybrid MXene/CNTs@SA fibers (Figure 3c), a more complex internal architecture is observed, where CNTs and MXene lamellae are randomly but intimately distributed. This intimate mixing suggests that CNTs may act as physical spacers to hinder MXene restacking and CNT agglomeration. In addition, EDS elemental mapping was also conducted on the hybrid fibers to evaluate the spatial distribution of each component. As shown in Figure 3d, Ti (originating from MXene) is uniformly dispersed throughout the fiber cross-section, while the overall C signal exhibits the highest intensity, attributed to the combined contributions from CNTs, SA, and MXene. More importantly, the homogeneous distribution of Na (from SA) indicates the uniform presence of the SA binder within the fiber matrix. These elemental observations, combined with the lamellar morphology observed by SEM, are consistent with the intercalation structure previously reported [28,29], wherein SA molecules intercalate into MXene.

Beyond the morphological and elemental evidence, FTIR was used to probe the interfacial chemical interactions among the components. As shown in Figure S2, the SA binder exhibits characteristic absorption bands at 3440 cm^−1^ (O-H stretching), 1633 cm^−1^ (asymmetric C=O stretching of carboxylate groups), 1418 cm^−1^ (C-O-H bending/C-C stretching), and 1025 cm^−1^ (C-O-C stretching), confirming the presence of abundant oxygen-containing functional groups. In the MXene@SA spectrum, an obvious red-shift in the O-H stretching peak is observed, indicating the formation of hydrogen bonding between the Ti-OH terminals on MXene surfaces and the hydroxyl/carboxyl groups of SA. Additionally, a new peak emerges at 558 cm^−1^ in both the MXene@SA and MXene/CNTs@SA spectra, which is assigned to the Ti-O vibration, a characteristic fingerprint of MXene, thereby confirming the successful incorporation of MXene. More significantly, the -COO^-^ asymmetric stretching peak at ~1633 cm^−1^ exhibits a detectable shift, suggesting coordination interactions or hydrogen bonding between the Ti atoms on MXene and the carboxylate groups of SA. These spectroscopic features collectively indicate that hydrogen bonds exist between MXene and SA, which stabilizes the hybrid structure and ensures fiber processability. Such a hierarchical architecture, combining chemical interfacial bonding (MXene–SA) and physical reinforcement (CNTs), is expected to facilitate efficient load transfer and charge transport within the final composite fibers.

### 3.3. Mechanical Properties

Mechanical properties are one of the most critical performance indicators for practical applications. Therefore, this study first compares the mechanical performance of macroscopic fibers prepared from various nanomaterials, as illustrated in Figure 4. The typical stress–strain curves of the MXene@SA, CNTs@SA, and MXene/CNTs@SA fibers are shown in Figure 4a. It can be observed that the fracture of the MXene@SA fiber is closer to brittle fracture. The brittle behavior of the MXene@SA fibers can be primarily attributed to the combined effects of MXene’s lower intrinsic strength relative to CNTs, structural disorder within the fiber, weak interlayer bonding, and a lack of effective load-transfer bridges [30]. However, the CNTs@SA, and MXene/CNTs@SA fibers undergo two distinct deformation stages during tensile testing. The first stage is the initial linear region, also referred to as the elastic region, in which the stress is directly proportional to the strain. In this stage, the fibers deform elastically and can return to its original shape upon unloading. Once the applied stress exceeds the yield point, the fibers enter the plastic deformation region, where permanent deformation occurs. The stress increases more slowly with strain or remains relatively constant. Finally, when the maximum tensile stress is reached, the fibers fracture, marking the end of the tensile process. CNTs inherently possess high tensile strength, and their fiber form typically exhibits plastic fracture behavior [31]. The incorporation of CNTs into MXene@SA fibers significantly enhances the strength and modulus of the resulting MXene/CNTs@SA composite fibers. This improvement is primarily attributed to three synergistic factors: (i) the intrinsically high mechanical strength of CNTs; (ii) their proposed role as physical “spacers” that intercalate between MXene nanosheets, which may help mitigate the restacking and dense aggregation of the sheets; and (iii) the formation of a hierarchical synergistic reinforcement network constructed from 1D CNTs and 2D MXene nanosheets [25]. The tensile strength of the CNT@SA fibers is considerably lower than that of the MXene/CNTs@SA fibers, primarily attributable to the intrinsic tendency of CNTs to aggregate and cross-link within the spinning stock solution, thereby forming large bundles that cannot be uniformly dispersed. Furthermore, the discontinuous arrangement and irregular orientation of CNTs within the fiber interior further compromise the efficiency of stress transfer, rendering the fibers susceptible to premature failure under relatively low applied loads [32]. In the present system, SA is introduced as a spinning aid, which is capable of establishing extensive hydrogen-bonding networks with MXene nanosheets and interacting with CNTs, thereby effectively improving the spinnability and structural homogeneity of the resulting fibers, as Figure 4d displays. Meanwhile, 2D MXene lamellae and 1D CNTs are proposed to serve mutually as physical “spacers” and “bridging agents” intercalated within the composite architecture (Figure 4c). This synergistic interplay may help suppresses the severe aggregation of CNTs and the restacking of MXene sheets, while also forming a hierarchical reinforcement network composed of 1D CNTs and 2D MXene [10,33]. Consequently, more efficient load transfer and stress distribution are achieved across the composite fiber, culminating in a substantial simultaneous enhancement in tensile strength.

Based on the stress–strain curves, the tensile strength and modulus of all fibers were obtained. As shown in Figure 3b, the tensile strengths of the MXene@SA, CNTs@SA, and MXene/CNTs@SA fibers are 67.89 ± 1.27 MPa, 50.55 ± 6.22 MPa, and 76.38 ± 7.09 MPa, respectively. Similarly, the corresponding Young’s modulus values, as presented in Figure 3c, are 11.71 ± 0.24 GPa, 2.25 ± 0.07 GPa, and 4.29 ± 0.38 GPa, respectively.

### 3.4. Electrical Properties

Given the critical role of electrical conductivity in enabling advanced applications (e.g., sensing, shielding, and energy storage), we compared the conductive performance of three fibers derived from distinct nanomaterials. As shown in Figure 3d, the electrical conductivities of the MXene@SA, CNTs@SA, and MXene/CNTs@SA fibers were measured to be 13.71 ± 0.45 S·cm^−1^, 26.23 ± 0.91 S·cm^−1^, and 16.14 ± 0.36 S·cm^−1^, respectively. Among the three types, the MXene@SA fibers exhibited the lowest conductivity, indicating that the electrical performance of MXene fibers fabricated via the current wet-spinning approach remains suboptimal and requires further improvement. Although monolayer MXene can exhibit conductivities in the range of 10^2^–10^4^ S·cm^−1^, assembling it into macroscopic fibers often leads to a substantial reduction. This decline is primarily attributed to the inclusion of additives (e.g., surfactants, sodium alginate, water), as well as structural factors possibly including interlayer restacking, defects, and poor orientation during the spinning process. In contrast, the CNTs@SA fibers exhibited a relatively higher conductivity of approximately 26.23 S·cm^−1^. The multi-walled CNTs employed in this work are of industrial grade, with an intrinsic conductivity exceeding 100 S·cm^−1^. The retention of a moderately high conductivity after fiber formation demonstrates that the wet-spinning method, combined with the calcium-crosslinked SA system, is effective for fabricating CNT-based fibers. To exploit the synergistic advantages of different nanomaterials, MXene/CNTs@SA composite fibers were fabricated by integrating two distinct nanoscale components. The incorporation of tubular CNTs into the lamellar architecture of MXene enhanced the electrical conductivity relative to the MXene@SA fibers and improved spinnability. Specifically, CNTs are proposed to act as physical spacers between adjacent MXene nanosheets, which may help mitigate the restacking of MXene layers and provide additional conductive pathways for efficient electron transport. As a result, the electrical conductivity of the MXene/CNTs@SA composite fibers was substantially improved compared to that of the pristine MXene fibers, confirming the beneficial role of CNT incorporation in optimizing the conductive performance.

### 3.5. Electrothermal Properties

To comprehensively evaluate the functional performance of the fabricated fibers, their electrothermal behavior was further characterized using a fiber-shaped Joule heating configuration. For practical applications, high electrical conductivity is essential to ensure safe operating voltages, portability, and rapid thermal responsiveness. In this study, a direct-current power supply at 12 V was applied across 2 cm-long fiber samples, and the surface temperature evolution was monitored in real time using an infrared thermal imaging camera. Upon application of an electric field, Joule heating arises from inelastic collisions between charge carriers and phonons within the fiber matrix [34,35]. The resulting steady-state saturation temperature (Ts) is governed by the dynamic equilibrium between Joule heat generation and heat dissipation to the surrounding environment. Figure 5a,d present the real-time temperature evolution of the three types of fibers under an identical applied voltage, while the corresponding thermal infrared images offer a more detailed and comprehensive visualization of the surface temperature distribution during electrothermal heating. As summarized in Figure 5b, the saturation temperatures (Ts) of the MXene@SA, CNT@SA, and MXene/CNTs@SA fibers reached 47.24 °C, 55.78 °C, and 52.77 °C, respectively. Correspondingly, their power densities (P_s_) were calculated to be 1.94 kW·cm^−3^, 3.39 kW·cm^−3^, and 2.95 kW·cm^−3^, respectively (Figure 5c).

The observed trend in Joule heating performance is qualitatively consistent with the independently measured electrical conductivity, confirming that superior charge transport facilitates more efficient electrothermal conversion The lower electrothermal efficiency of the pure MXene fibers is ascribed to their limited conductivity arising from structural disorder and interlayer resistance, whereas the incorporation of CNTs into the MXene matrix effectively improves both charge transport and heat generation, as reflected by the intermediate performance of the MXene/CNTs@SA composite fibers. These results demonstrate that the MXene/CNTs@SA composite fibers offer a balanced trade-off between processability, conductivity, and electrothermal capability, making them promising candidates for flexible and wearable electronic applications.

### 3.6. Flexibility and Sensing Performance

Periodic bending tests were performed on the MXene/CNTs@SA fibers at various bending angles. Figure 6a presents the ΔR/R_0_ response of the composite fiber during a complete bending–releasing cycle, plotted as a function of time. With increasing applied displacement, the fibers were subjected to a range of bending angles, and ΔR/R_0_ was observed to increase monotonically with the bending angle. This trend can be ascribed to the greater mechanical strain imposed by larger bending angles, which induces more pronounced restructuring of the internal conductive network. Under bending, microstructural deformations within the fibers disrupt the electron transport pathways, thereby causing the resistance change.

To evaluate the long-term mechanical-electrical stability, extended cycling tests were carried out over 100 continuous bending-release cycles. As shown in Figure 6b, the ΔR/R_0_ response curves exhibit highly consistent signal amplitude and waveform throughout the entire process, with the baseline resistance drift remaining below 1.6% after 100 cycles. This minimal variation not only confirms the outstanding electrical reproducibility of the fibers but also indirectly demonstrates that no substantial interfacial fatigue or debonding occurs between the SA matrix and the conductive fillers during prolonged cycling, as such degradation would otherwise result in an irreversible increase in resistance.

Upon release of the bending load, the internal structure rapidly recovered, and ΔR/R_0_ returned to its initial baseline. This excellent reversibility is attributed to the wet-spinning fabrication process, which ensures firm and uniform integration of MXene and CNT sheets within the fiber matrix, as opposed to superficial surface coating [36]. To quantitatively evaluate the strain sensing sensitivity, four different bending states were applied (Figure 6c), and the GF was calculated using the formula 4–6. The GF values obtained from the four states are 7.12, 7.00, 7.52, and 7.80, respectively, yielding an average GF of 7.36 (Figure 6c), which is comparable to or even higher than many recently reported fiber-based strain sensors and is well sufficient for practical motion monitoring applications [37]. Collectively, these results confirm that the MXene/CNTs@SA fibers combine high sensitivity with outstanding cyclic stability in bending sensing, providing a solid foundation for their potential applications in flexible electronic devices and wearable sensors.

## 4. Conclusions

In this study, sodium alginate was employed as both a binder and a fiber-forming agent to fabricate MXene@SA, CNTs@SA, and their composite nanocomposite fibers via wet spinning. The effects of different nanomaterial combinations on the microstructure and overall performance of the resulting fibers were systematically investigated. SEM characterization revealed that all fibers exhibited longitudinal surface wrinkles and a nearly circular cross-section, indicating good controllability of the spinning process. Compared with the pure MXene@SA fibers, the incorporation of CNTs constructed a hierarchical synergistic reinforcing network through the synergistic interplay between 1D CNTs and 2D MXene nanosheets, which effectively suppressed MXene restacking and enhanced structural uniformity. Thus, the MXene/CNTs@SA fibers got the highest tensile strength among all compositions, which was 12.51% and 51.10% higher than that of the MXene@SA and CNTs@SA fibers, respectively. Meanwhile, the electrical conductivity and Joule heating performance of the MXene/CNTs@SA fibers were substantially improved relative to the MXene@SA fibers, benefiting from the additional conductive pathways provided by CNTs. Notably, the CNTs@SA fibers retained superior electrical conductivity and electrothermal performance due to the intrinsic high conductivity of CNTs and their continuous conductive network, reflecting an inherent trade-off between mechanical robustness and electrical functionality. Further evaluation of the flexibility and sensing behavior of the MXene/CNTs@SA fibers demonstrated that, under 100 cycles of repeated bending, the resistance change stayed below ± 1.6%, underscoring their excellent flexibility and recovery capability. The bending sensitivity (GF) of the MXene/CNTs@SA fibers was determined to be 7.36. Therefore, these results indicate that MXene/CNTs@SA composite fibers hold great promise for applications in flexible electronic devices and wearable sensors.

## Figures and Tables

**Figure 1:**
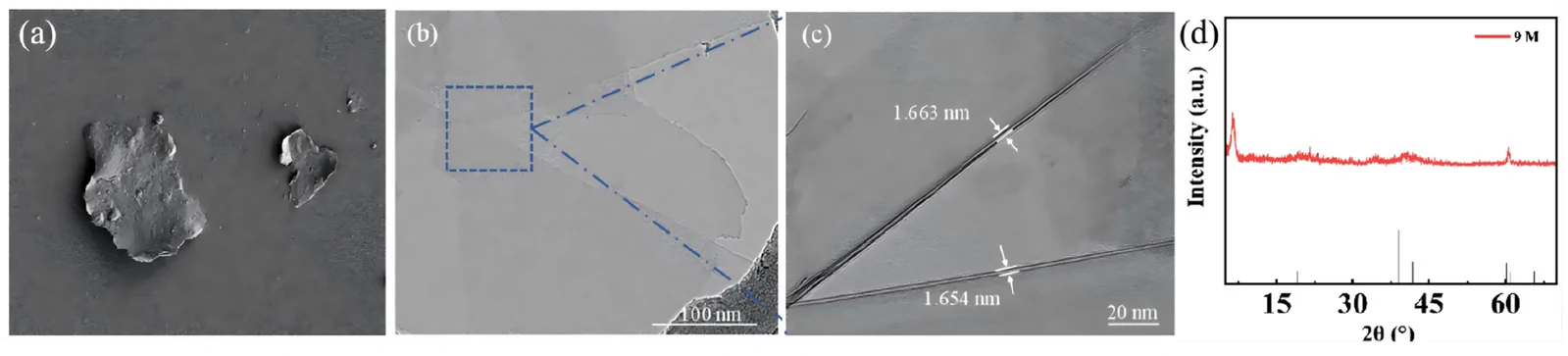
The microstructure of MXene. (**a**) The SEM image of MXene; (**b**) the TEM image of MXenes; (**c**) the HRTEM images of MXene; (**d**) the XRD curve of prepared MXene.

**Figure 2:**
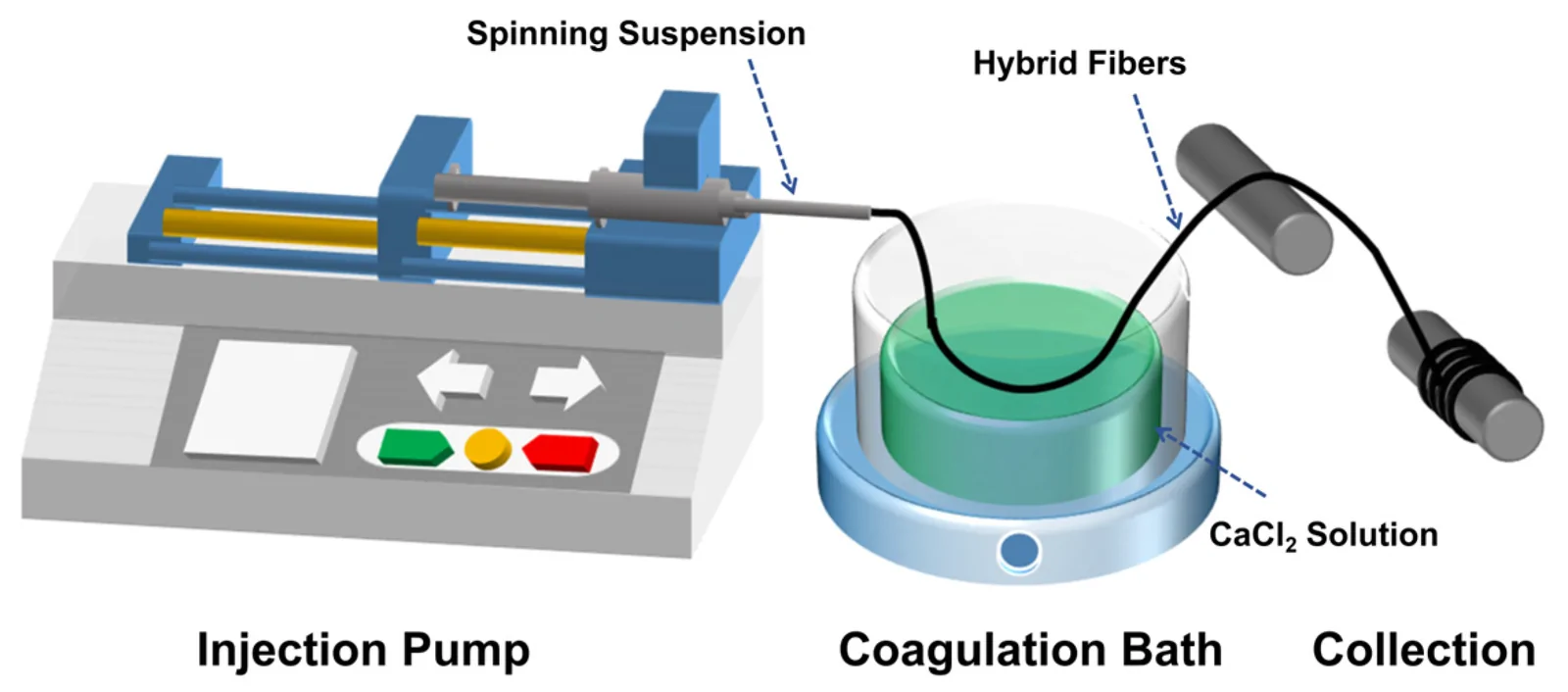
A schematic diagram showing the fabrication process of the fibers.

**Figure 3:**
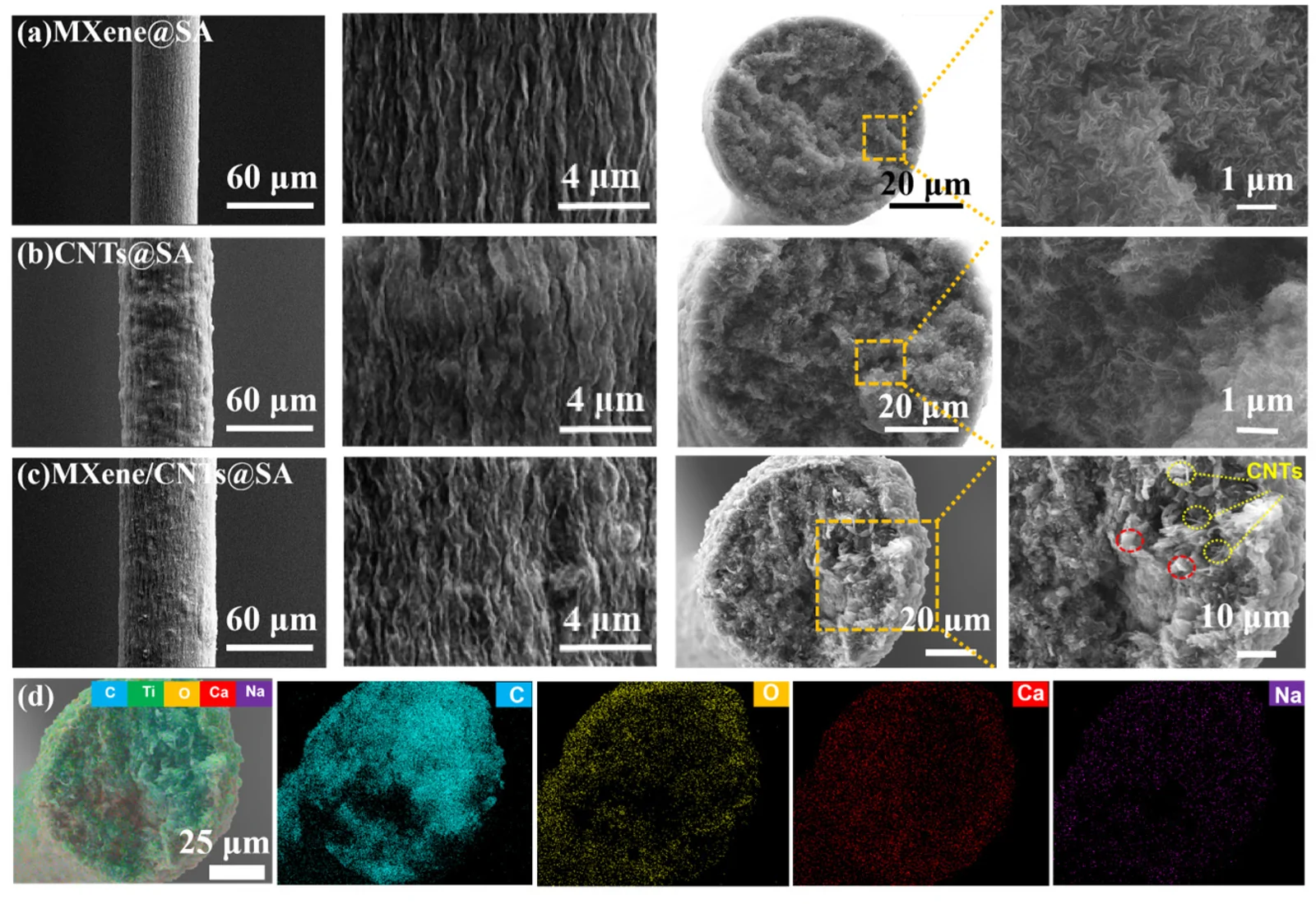
SEM images of fiber morphologies and cross-sections of different fibers. (**a**) MXene@SA fibers; (**b**) CNTs@SA fibers; (**c**) MXene/CNTs@SA fibers; (**d**) EDS results of MXene/CNTs@SA fibers.

**Figure 4:**
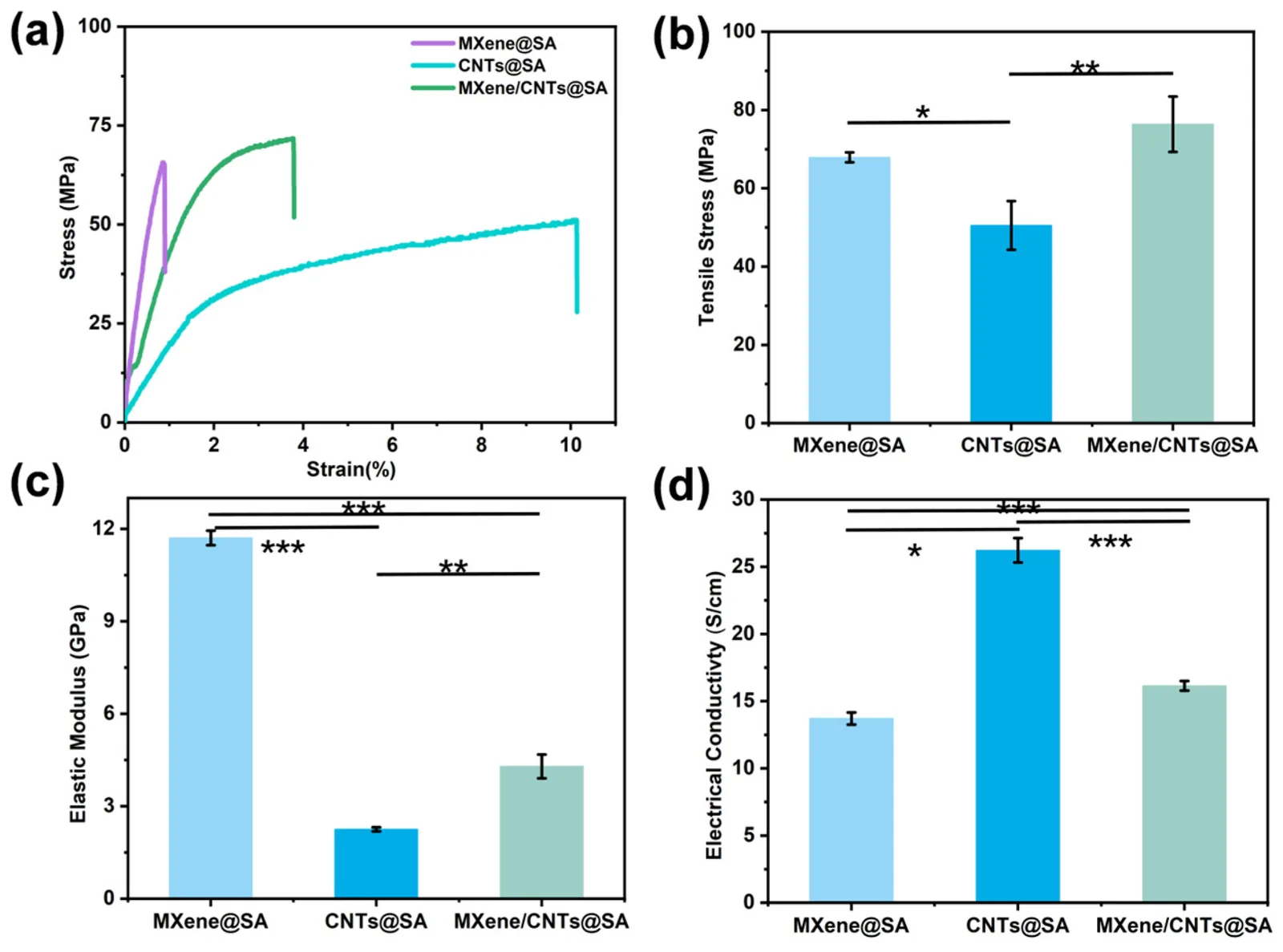
Mechanical and electronical properties of different composite fibers. (**a**) Typical stress−strain curves; (**b**) tensile strength; (**c**) tensile modulus; (**d**) electrical conductivity. Results are presented as means ± standard deviations (n = 3; * *p* < 0.05, ** *p* < 0.01, and *** *p* < 0.001).

**Figure 5:**
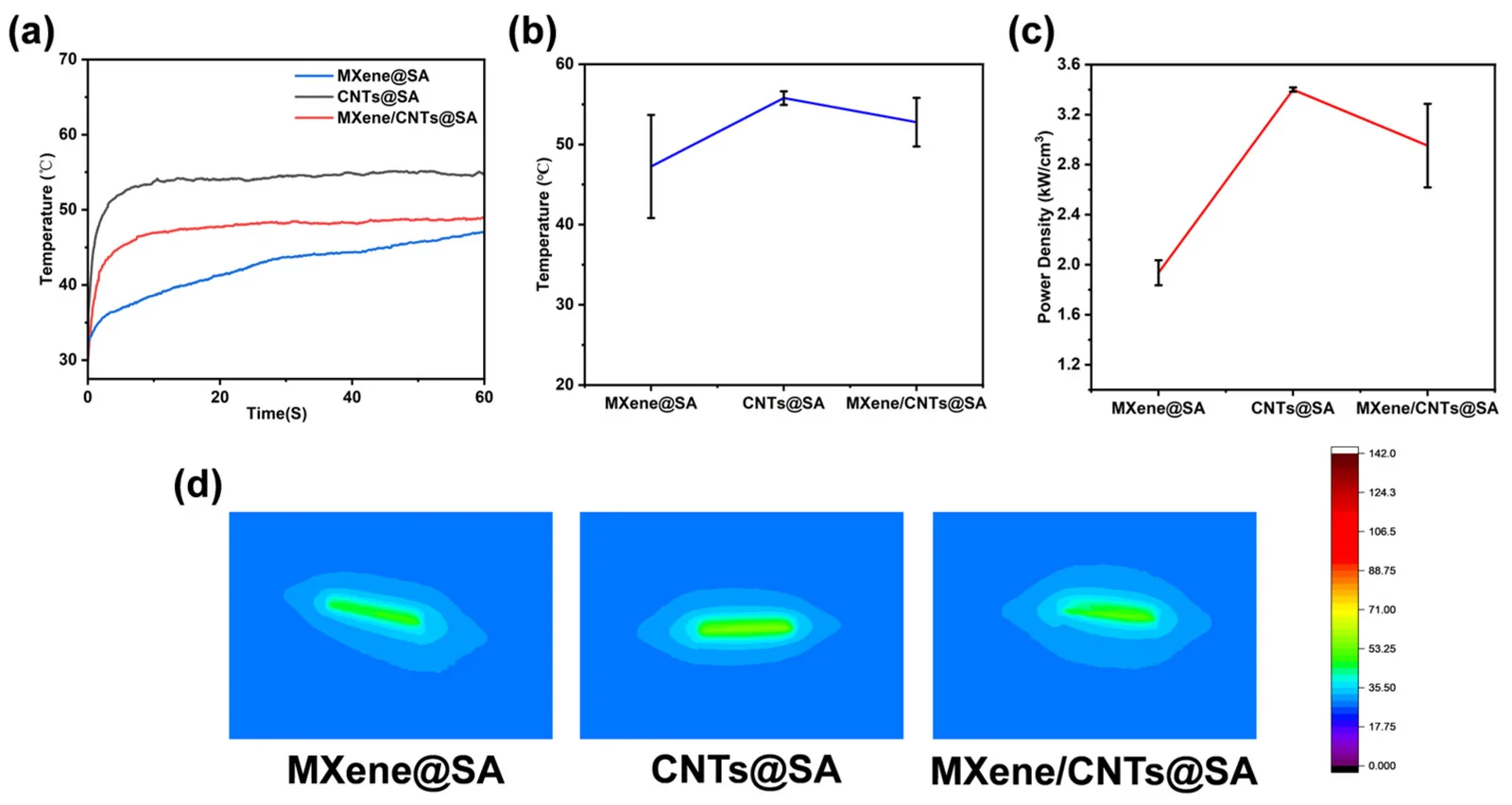
Electrothermal performance of fibers under 12 V. (**a**) Temperature evolution over time. (**b**) Saturation temperature. (**c**) Power density. (**d**) Thermal infrared images.

**Figure 6:**
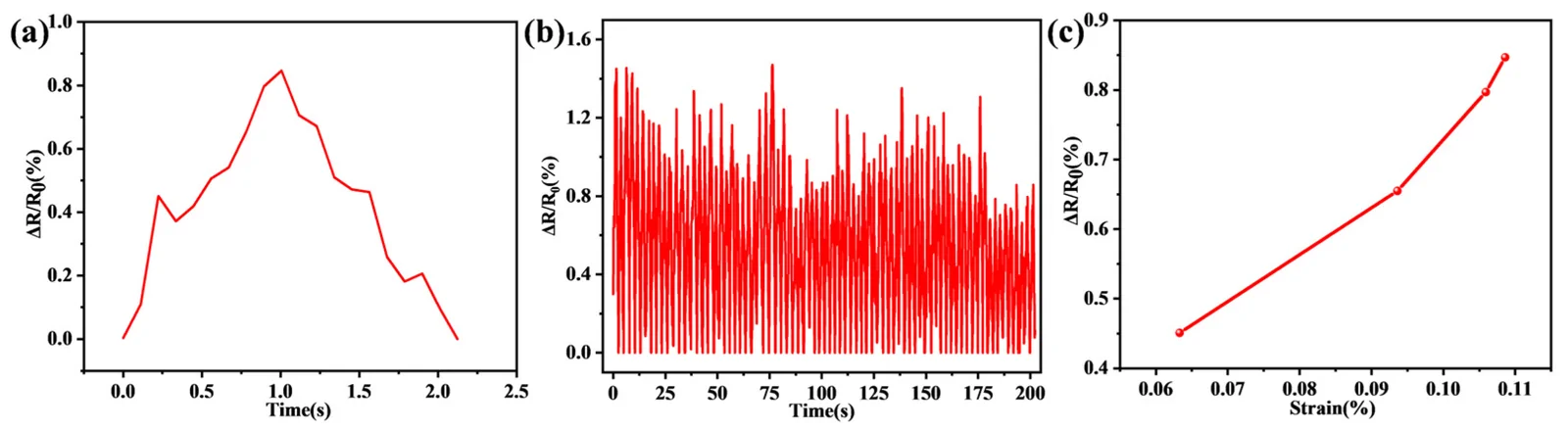
Sensing performance of MXene/CNTs@SA fibers: (**a**) resistance change curves at various bending angles; (**b**) resistance change curves under cyclic bending; (**c**) sensitivity.

## Data Availability

The raw data supporting the conclusions of this article will be made available by the authors on request.

## Supplementary Materials

The following supporting information can be downloaded at: https://www.mdpi.com/article/10.3390/bios16100532/s1, Figure S1: Photographs of MXene@SA composite fibers prepared with different mass ratios of SA; Figure S2: FTIR results of SA, CNTs@SA, MXene@SA and CNTs/MXene@SA fibers.

## References

[1] Hu Z., Geng J., Zhang Z., Ke H., Wang K., Xu S., Shang C., Qiao Y., Xiao L., Bai C. (2026). Design strategies of MXene-enabled two-dimensional flexible force sensors: From sensing architectures to scalable assembly. Carbon.

[2] Du T., Han X., Yan X., Shang J., Li Y., Song J. (2023). MXene-Based Flexible Sensors: Materials, Preparation, and Applications. Adv. Mater. Technol..

[3] Jiang J., Chen X., Niu Y., He X.-R., Hu Y.-L., Wang C. (2022). Advances in flexible sensors with MXene materials. New Carbon Mater..

[4] Naguib M., Kurtoglu M., Presser V., Lu J., Niu J., Heon M., Hultman L., Gogotsi Y., Barsoum M.W. (2011). Two-Dimensional Nanocrystals Produced by Exfoliation of Ti_3_AlC_2_. Adv. Mater..

[5] Shang C., Wang F., Gu Y., Sun G., Qiao Y., Shen J., Zheng T., Wang X. (2025). Interfacial Properties Prediction of Carbon Fiber Composites via Multiscale Nanomaterial Enhancement and Wetting Parameter Analysis. Polym. Compos..

[6] Zhang G.-L., Du Y.-Q., Guo Y.-T., Liu H., Wang L.-B., Jiang J.-Z., Zhou A.-G. (2025). Preparation of Mo2CTx MXene with high specific surface area by etching of acetic acid/acetate solution. J. Cent. South Univ..

[7] Shang C., Nan H., Bai C., Zhang L., Sun G., Qiao Y., Wang X., Shen J., Zheng T. (2025). The PEEK sizing agents modified by different dimensions nanomaterials of MXene and CNTs for enhancing the interfacial and mechanical properties of carbon fiber composites. Colloids Surf. A Physicochem. Eng. Asp..

[8] Jin L., Ji Y., Li Y. (2025). Ten-Electron Count Rule of MXene-Supported Single-Atom Catalysts for Sulfur Reduction in Lithium–Sulfur Batteries. Carbon Neutraliz..

[9] Su H., Jin C., Zhang X., Yu Z., Zeng X. (2024). Recent progress in the synthesis and electrocatalytic application of MXene-based metal phosphide composites. Carbon Neutraliz..

[10] Kumar J.A., Prakash P., Krithiga T., Amarnath D.J., Premkumar J., Rajamohan N., Vasseghian Y., Saravanan P., Rajasimman M. (2022). Methods of synthesis, characteristics, and environmental applications of MXene: A comprehensive review. Chemosphere.

[11] Chen K.-F., Cai P., Peng H.-L., Xue X.-G., Wang Z.-M., Sun L.-X. (2021). Ti3C2Tx MXene for organic/perovskite optoelectronic devices. J. Cent. South Univ..

[12] Xu S., Zhang X., Zheng T., Zhao Z., Shang C., Hu Z., Dong M., Qiao Y., Bai C., Zhang X. (2025). Advancements in high-performance MXene composite fibers integrated with various functional materials: Fabrication, functionalization, property enhancement, and applications. J. Mater. Sci. Technol..

[13] Li Y., Zhang X. (2021). Electrically Conductive, Optically Responsive, and Highly Orientated Ti_3_C_2_T*_x_* MXene Aerogel Fibers. Adv. Funct. Mater..

[14] Dong M., Ke H., Wang F., Wang K., Sun G., Zheng T., Wang X. (2026). High-strength, electrochemically efficient RuO2-MXene/CNTs hybrid fibres via nanostructure design for flexible electronics and energy storage. Mater. Des..

[15] Fu X., Yang H., Li Z., Liu N.C., Lee P.S., Li K., Li S., Ding M., Ho J.S., Li Y.C.E. (2023). Cation-induced assembly of conductive MXene fibers for wearable heater, wireless communication, and stem cell differentiation. Sci. Eng..

[16] Dong L., Ren M., Wang Y., Yuan X., Wang X., Yang G., Li Y., Li W., Shao Y., Qiao G. (2024). Sodium alginate-based coaxial fibers synergistically integrate moisture actuation, length tracing, humidity sensing, and electric heating. Mater. Horiz..

[17] Sun S., Zhu X., Wu X., Xu M., Hu Y., Bao N., Wu G. (2022). Covalent-architected molybdenum disulfide arrays on Ti3C2Tx MXene fiber towards robust capacitive energy storage. J. Mater. Sci. Technol..

[18] Qiu H., Qu X., Zhang Y., Chen S., Shen Y. (2023). Robust PANI@MXene/GQDs-Based Fiber Fabric Electrodes via Microfluidic Wet-Fusing Spinning Chemistry. Adv. Mater..

[19] Lai J., Yu Y., Zhang X., Qiang W., Zhang X. (2024). Interlaminar fracture toughness and impact resistance of carbon fiber reinforced composite with magnetic aligned CNTs. Compos. Part B Eng..

[20] Huang P., Yang L., Wang F., Fu X., Hu C., Yang B. (2024). Highly sensitive and stretchable fiber strain sensors based on ITO NPs/CNTs composite. Measurement.

[21] Zheng T., Wang X., Liu Y., Bayaniahangar R., Li H., Lu C., Xu N., Yao Z., Qiao Y., Zhang D. (2020). Polyaniline-decorated hyaluronic acid-carbon nanotube hybrid microfiber as a flexible supercapacitor electrode material. Carbon.

[22] Zhu Y., Rao B., Zhao Z., Zheng T., Qu W., Wang X., Liu Y., Bai C., Colombo P. (2025). Production of continuous carbon nanotube/geopolymer composite fibers by wet spinning for adsorption applications. Eur. Phys. J. Plus.

[23] Wang W., Guo W., Zhu J., Li H., Liu S., Erden F., Li P., Yang M., He C. (2025). Highly conductive wet-spun PANI/CNTs fibers for wearable energy harvesting and thermal sensing. J. Colloid Interface Sci..

[24] Ahmed A.Y., Shoukat R., Ali A., Muqaddas S., Bayach I., Alsadun N. (2025). Polyoxometalate modified CNTs fiber electrode as efficient electrocatalyst towards water splitting reactions. Polyhedron.

[25] Zhao X., Zhang J., Lv K., Kong N., Shao Y., Tao J. (2022). Carbon nanotubes boosts the toughness and conductivity of wet-spun MXene fibers for fiber-shaped super capacitors. Carbon.

[26] Zheng T., Zhang X., Li Y., Zhu Y., Yan W., Zhao Z., Zhang L., Bai C., Wang X. (2023). Wet-spinning of continuous hyaluronic-based MXene/CNTs hybrid fibers for flexible supercapacitor applications. Mater. Lett..

[27] Sa V., Kornev K.G. (2011). A method for wet spinning of alginate fibers with a high concentration of single-walled carbon nanotubes. Carbon.

[28] Hou K., Zhou H., Zhu K., Xie C., Liu S., He Y., Zhu X., Wu M., Huang T. (2022). Superwetting Ti3C2TX MXene membranes intercalated with sodium alginate for oil/water separation. Carbohydr. Polym. Technol. Appl..

[29] Wang G., Zhang R., Zhang H., Cheng K. (2023). Aqueous MXene inks for inkjet-printing microsupercapacitors with ultrahigh energy densities. J. Colloid Interface Sci..

[30] Zheng Y., Wang Y., Zhao J., Li Y. (2023). Electrostatic Interfacial Cross-Linking and Structurally Oriented Fiber Constructed by Surface-Modified 2D MXene for High-Performance Flexible Pseudocapacitive Storage. ACS Nano.

[31] Bai Y., Zhang R., Ye X., Zhu Z., Xie H., Shen B., Cai D., Liu B., Zhang C., Jia Z. (2018). Carbon nanotube bundles with tensile strength over 80 GPa. Nat. Nanotechnol..

[32] Wang Z., Qin S., Seyedin S., Zhang J., Wang J., Levitt A., Li N., Haines C., Ovalle-Robles R., Lei W. (2018). High-Performance Biscrolled MXene/Carbon Nanotube Yarn Supercapacitors. Small.

[33] Zhou Z., Panatdasirisuk W., Mathis T.S., Anasori B., Lu C., Zhang X., Liao Z., Gogotsi Y., Yang S. (2018). Layer-by-layer assembly of MXene and carbon nanotubes on electrospun polymer films for flexible energy storage. Nanoscale.

[34] Zhang J., Uzun S., Seyedin S., Lynch P.A., Akuzum B., Wang Z., Qin S., Alhabeb M., Shuck C.E., Lei W. (2020). Additive-Free MXene Liquid Crystals and Fibers. ACS Cent. Sci..

[35] Luo J., Lu H., Zhang Q., Yao Y., Chen M., Li Q. (2016). Flexible carbon nanotube/polyurethane electrothermal films. Carbon.

[36] Xiao W., Wu H., Lu L., Yan J., Huang X., Wang L., Wang J., Gao J. (2026). MXene-mediated interfacial design of Janus conductive fiber composites for synergistic multifunctional wearable sensing. Compos. Part A Appl. Sci. Manuf..

[37] Hajian S., Maddipatla D., Narakathu B.B., Atashbar M.Z. (2022). MXene-based flexible sensors: A review. Front. Sens..

