# Supplementary material for: Multifunctional MXene/CNTs Composite Fibers with High Strength, Conductivity, and Bending Sensitivity Fabricated via Sodium Alginate-Assisted Wet Spinning
Source: Biosensors (Basel). 2026 Sep 24;16(10):532. doi: 10.3390/bios16100532 (PMC13702720; doi:10.3390/bios16100532)
Supplement: Supplementary file 1 [file biosensors-16-00532-s001.zip › biosensors-4509534-supplementary.pdf]

Type of the Paper (Article)

Multifunctional MXene/CNTs Composite Fibers with High Strength, Conductivity, and Bending Sensitivity Fabricated via Sodium Alginate-Assisted Wet Spinning

Ting Zheng [1,2], Shuting Xu [2], Zherui Zhang[2], Kaixuan Wang[2], Xiaodong Wang[2,*] and Guoxing Sun [1,*]

1 Joint Key Laboratory of the Ministry of Education, Institute of Applied Physics and Materials Engineering, University of Macau, Avenida da Universidade, Taipa, Macau SAR, 999078, China; tingzheng@um.edu.mo
2 Key Laboratory of Superlight Materials and Surface Technology, Ministry of Education, College of Materials Science and Chemical Engineering, Harbin Engineering University, Harbin, 150001, China
* Correspondence: wangxiaodong@hrbeu.edu.cn (X.W.); gxsun@um.edu.mo (G.S.)

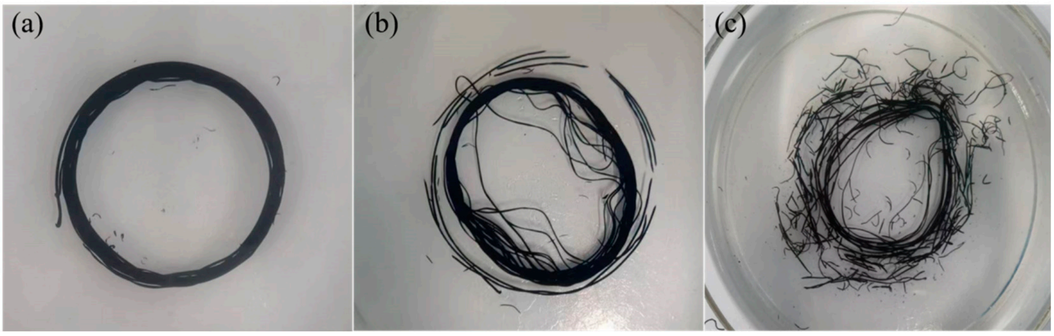

Figure S1. Photographs of MXene@SA composite fibers prepared with different mass ratios of SA. (a) 20 wt% (b) 15 wt% (c) 10 wt%

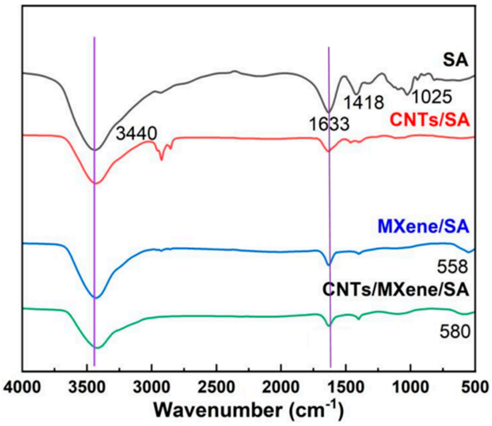

Figure S2. FTIR results of SA, CNTs@SA, MXene@SA and CNTs/MXene@SA fibers.
